# Gastric Trichobezoar Causing Intermittent Small Bowel Obstruction: Report of a Case and Review of the Literature

**DOI:** 10.1155/2011/217570

**Published:** 2011-06-07

**Authors:** Nicole G. Coufal, Akash P. Kansagra, Jay Doucet, Jeanne Lee, Raul Coimbra, Vishal Bansal

**Affiliations:** ^1^University of California San Diego, School of Medicine, 9500 Gilman Drive, La Jolla, San Diego, CA 92093, USA; ^2^Department of Radiology & Biomedical Imaging, University of California San Francisco, 505 Parnassus Avenue, M-391, San Francisco, CA 94143, USA; ^3^Department of Surgery, University of California San Diego, 9500 Gilman Drive, San Diego, CA 92093, USA; ^4^Division of Trauma, Surgical Critical Care and Burns, University of California, San Diego School of Medicine, 9500 Gilman Drive, San Diego, CA 92093, USA

## Abstract

We report the unusual case of a 45-year-old woman who presented with multiple episodes of small bowel obstruction. Initial exploratory lap-roscopy did not reveal an etiology of the obstruction. Subsequent upper endoscopy identified a non-obstructing gastric trichobezoar which could not be removed endoscopically but was not thought to be responsible for the small bowel obstruction given its location. One week postoperatively, the patient experienced recurrence of small bowel obstruction. Repeat endoscopy disclosed that the trichobezoar was no longer located in the stomach and upon repeat laparotomy was extracted from the mid-jejunum. In the following 8 months, the patient had no further episodes of small bowel obstruction. Consequently, gastric bezoars should be included in the differential diagnosis of recurrent small bowel obstruction.

## 1. Introduction

Trichobezoars are collections of hair which accumulate and remain within the gastrointestinal tract for extended periods. They occur so rarely that their true incidence is unknown [1]. These bezoars are typically found in the stomach but may also occur in the small or large bowel. The associated constellation of symptoms correlates to the precise location of the bezoar. We report here an unusual case of an endoscopically confirmed gastric trichobezoar without small bowel extension that nonetheless presented with clinical and radiographic signs of recurrent, intermittent small bowel obstruction and was later found to have migrated to the jejunum.

## 2. Case Report

**Figure 1:**
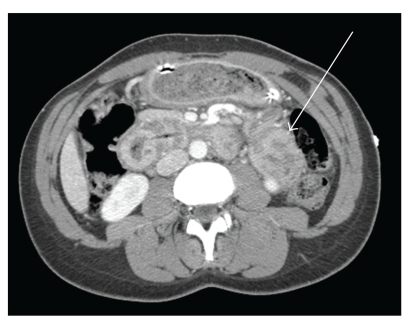
CT at initial presentation indicating intussusception of a short segment of small bowel.

A 45-year-old African American woman presented to our institution with two months of episodic, colicky, epigastric pain culminating in two days of nausea, vomiting, and severe abdominal pain. The patient also reported absence of bowel movements for two weeks in addition to an intermittently palpable epigastric mass. She had a history of small bowel obstruction two years prior which had been successfully managed by nonoperative means, including nasogastric decompression. Physical examination disclosed mild epigastric tenderness but no palpable mass. On admission, a complete blood count was notable for microcytic anemia, with a hemoglobin of 8.8 gm/dL, a mean corpuscular volume (MCV) of 62.7 *μ*m^3^, a mean corpuscular hemoglobin (MCH) of 18.4 pgm, and a mean corpuscular hemoglobin concentration (MCHC) of 29.3% but otherwise normal. Notably, her red cell distribution width (RDW) was elevated at 20.6%, and a peripheral smear was notable for mild hypochromia. A differential showed 82% neutrophils and 10% lymphocytes but no left shift. A basic metabolic panel, liver function tests, and lipase were found to be within normal limits, with a normal albumin of 4.5 gm/dL. Urinalysis was only notable for small ketones. Abdominal plain films demonstrated dilated loops of small bowel and multiple air-fluid levels, consistent with a diagnosis of small bowel obstruction. Subsequent computed tomography (CT) of the abdomen disclosed signs of jejunal intussusception (Figure 1).

**Figure 2:**
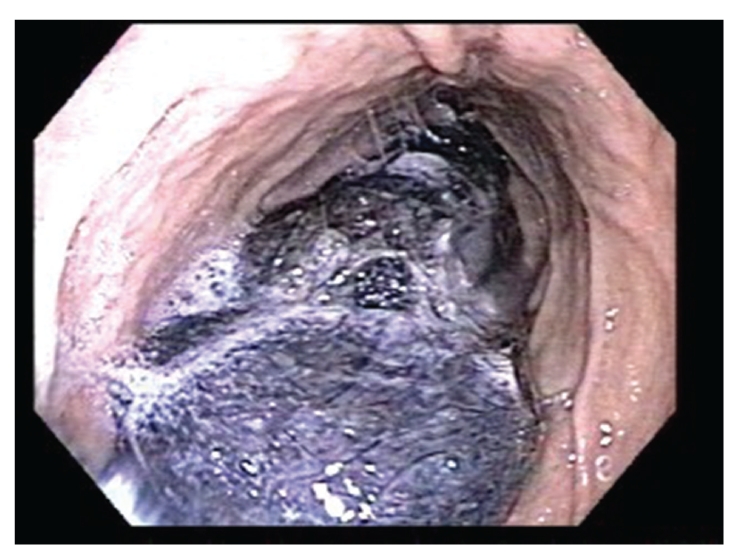
Upper endoscopy following initial laparoscopy demonstrating a nonobstructing gastric trichobezoar.

Based on radiographic findings and the patient's history of recurrent small bowel obstruction, the decision was made to pursue exploratory laparoscopy. This procedure was performed without complication, but no cause of her small bowel obstruction could be identified. Of note, the intussuscepted segment of bowel noted on CT was not present intraoperatively, and peritoneal samples were negative for bacterial or fungal growth. Due to continued presence of symptoms following laparoscopy, upper endoscopy was performed to identify other possible causes of the patient's symptoms. Endoscopy disclosed a previously unknown large, nonobstructing trichobezoar confined to the stomach (Figure 2). Mucosal samples taken during endoscopy exhibited acute jejunitis with ulceration and reactive atypica. Though unusual, the presence of a nonobstructing gastric trichobezoar was thought to be an unlikely cause of obstructive symptoms in the small bowel. Numerous attempts to remove the material endoscopically with a Roth net and snare were unsuccessful. In the subsequent days, the patient improved clinically with conservative measures and was able to tolerate oral intake. In light of this improvement, more aggressive attempts to remove the trichobezoar were deferred.

**Figure 3:**
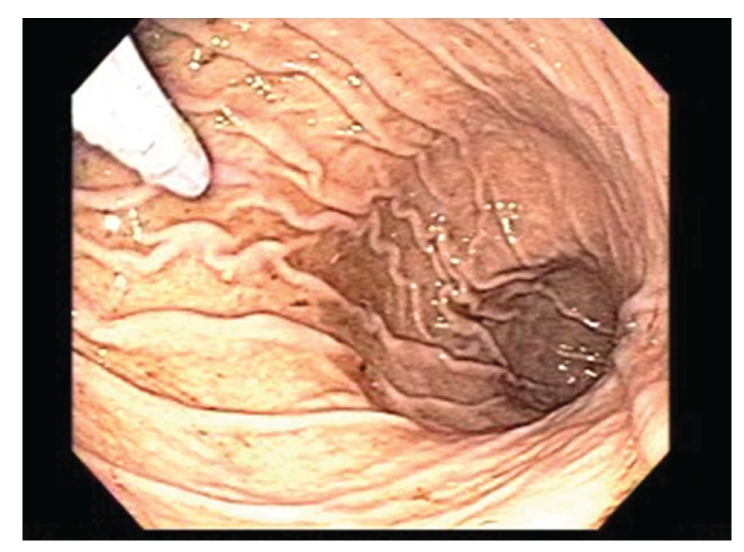
Upper endoscopy at the time of symptom recurrence exhibiting absence of the previously present gastric trichobezoar.

**Figure 4:**
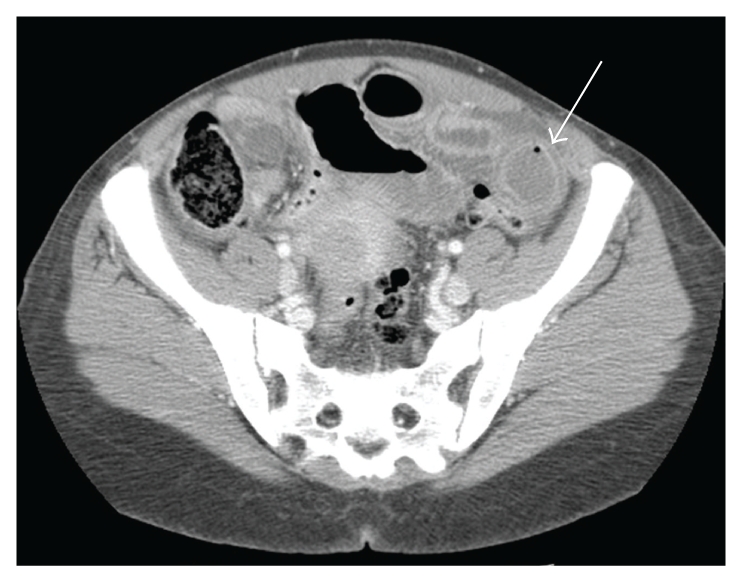
CT at the time of symptom recurrence exhibiting dilated small bowel with mural stratification.

**Figure 5:**
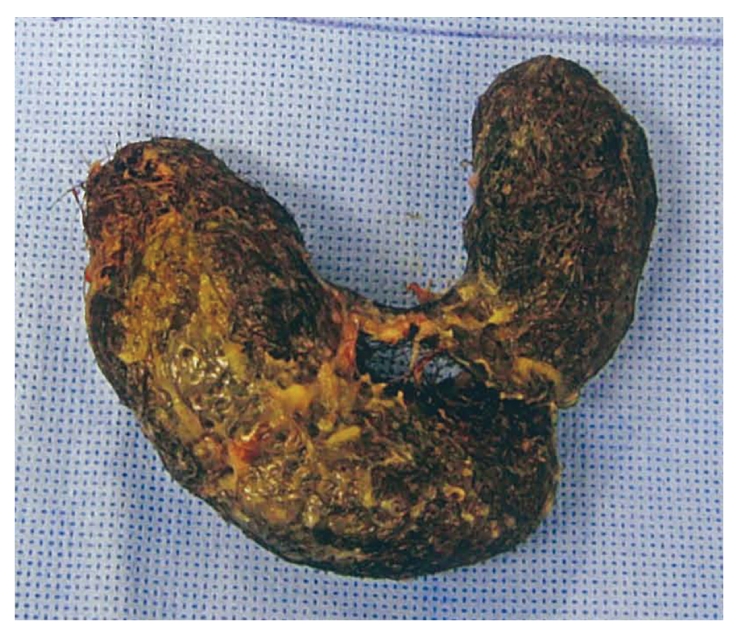
Large trichobezoar removed via laparotomy.

One week later, the patient reported a recurrence of symptoms, with abdominal pain, distension, and vomiting. Laboratory findings at the time of recurrence included a leukocytosis of 15,700 cells/mm^3^, anemia with hemoglobin of 8.9 gm/dL, albumin of 3.9 gm/dL, and normal electrolyte and hepatic enzyme levels. Repeat upper endoscopy found no evidence of the previously visualized trichobezoar in the stomach or duodenum (Figure 3). A CT of the abdomen exhibited dilated small bowel with mural stratification and inspissated material suggestive of moderate- to high-grade distal small bowel obstruction (Figure 4). The patient subsequently underwent uncomplicated midline laparotomy, resulting in the removal of a 13 × 3 cm trichobezoar from the mid-jejunum (Figure 5). The patient recovered uneventfully, with no recurrence of symptoms for the eight months of recorded followup. 

## 3. Discussion

Trichobezoars arise from the ingestion of hair, which is ineffectively moved by peristalsis due to its smooth surface and poorly digested keratinaceous substance. As a result, the hair becomes matted into a ball and is retained in the folds of gastric mucosa. The ball can reach sizes sufficient to cause stomach distension and inhibit gastric emptying. Trichobezoars occur more frequently in women, with only isolated cases reported in males [2], and occur most frequently between the ages of 13–20, with the oldest case being described in a 54-year-old [3]. Trichobezoars often coexist with learning disabilities or psychiatric illness [4].

Large bezoars are generally retained in the stomach, likely due to the presence of a sphincter at the pylorus. There, they may produce symptoms related to obstruction at this level, including nonbilious vomiting, dehydration, chronic failure to thrive, and colicky abdominal pain. The most common presenting symptoms include abdominal pain, nausea, and vomiting, which occur in 33–37% of patients [3]. Anemia and hypoalbuminemia have also been described [5]. A palpable mass in the upper abdomen, known as Lamerton's sign, may also be present [6]. Less commonly, small bowel trichobezoars may cause intestinal obstruction. There have also been extremely rare reports of gastric trichobezoars with “tails” that extend into the small bowel, the so-called Rapunzel syndrome, that cause symptoms of intestinal obstruction despite their predominantly gastric location. To our knowledge, only 24 such cases have been reported in the literature to date [3].

Potential complications of a gastric trichobezoar include failure to thrive, gastric ulceration, anemia, and hypoalbuminemia, as well as more acute findings of intestinal or pancreaticobiliary obstruction resulting in perforation, peritonitis, or pancreatitis [7]. Anemia and hypoalbuminemia are postulated to occur due to protein wasting and chronic gastritis resulting from gastric irritation by the bezoar [6, 7] and were first reported in 1921 in a case of trichobezoar presenting with six months of edema [8]. 

The case described here is especially unusual because it involves a gastric trichobezoar that caused intermittent small bowel obstruction despite the lack of a tail or any other portion extending into the small bowel. The precise mechanism by which an entirely gastric trichobezoar can cause small bowel obstruction is unclear. Intermittent migration of the trichobezoar into the small bowel may offer an explanation, supported in this case by the eventual migration of the trichobezoar into the jejunum and by existing evidence that bezoars may move in a retrograde direction [9]. This case highlights the importance of considering gastric bezoars as a possible cause of intermittent small bowel obstruction.

## 4. Conclusion

Gastric bezoars should be included in the differential diagnosis of recurrent small bowel obstruction. Despite their gastric location, they may cause symptoms of small bowel obstruction, possibly via intermittent migration into the small intestine. Removal of an incidental nonobstructing trichobezoar in a patient with symptoms of small bowel obstruction should be considered in the absence of other identifiable causes of obstruction, especially if these symptoms are recurrent.
